# Suicide decline and improved psychiatric treatment status: longitudinal survey of suicides and serious suicide attempters in Tokyo

**DOI:** 10.1186/s12888-022-03866-7

**Published:** 2022-03-29

**Authors:** Yasushi Otaka, Ryosuke Arakawa, Ryuichiro Narishige, Yoshiro Okubo, Amane Tateno

**Affiliations:** 1 Department of Neuropsychiatry, Graduate School of Medicine, Nippon Medical School, 1-1-5, Sendagi, Bunkyo-ku, Tokyo, 113-8602 Japan; 2 Wakamiya Hospital, Yamagata, Japan

**Keywords:** Longitudinal studies, Psychiatric treatment status, Serious suicide attempter, Death by suicide, Suicide decline in Japan

## Abstract

**Background:**

Connecting individuals in need of psychiatric treatment with adequate medical services has been a major strategy for suicide prevention in Japan. By investigating serious suicide attempters admitted to our Critical Care Medical Center (CCM), we aimed to examine longitudinal changes in the psychiatric treatment status of high-risk suicidal individuals, and to explore the association between any improvement in psychiatric treatment status and suicide decline.

**Methods:**

Subjects from two periods, 2006–2011 and 2012–2017, were enrolled. We collected the data of 32,252 suicides in Tokyo from police reports and the data of 942 suicide attempters admitted to CCM from medical records. Data were annually collected by both age and gender for the number of suicide completers, the number of suicide attempters, and the psychiatric treatment rates, respectively.

ANOVA and t-test were used to examine whether there were differences in the number of suicides and attempers between the two periods. The difference in psychiatric treatment rate between the two periods was examined by chi-square test. Additionally, we used Pearson’s correlation coefficient to analyze any correlation between annual treatment rate and the number of suicide completers in subgroups with altered psychiatric treatment rates.

**Results:**

The number of suicide attempters in the 20–39-year age group of decreased together with the number of suicides. Psychiatric treatment rates of male attempters aged 20–59 years improved significantly from 48.7 to 70.6% and this improvement correlated with a decrease in suicides. However, psychiatric treatment rates in the elderly, which have the highest number of suicides in both genders, did not improve and remain low.

**Conclusions:**

The number of suicide attempters, as well as that of suicides, decreased in Tokyo. Improvement of psychiatric treatment status in high-risk suicidal male adults may have contributed to the reduction of suicides in Tokyo. However, the continuing low rate of psychiatric treatment in the elderly is a pressing issue for future suicide prevention.

## Background

The number of suicides in Japan suddenly increased in 1998, and it remained at an annual rate of over 30,000, with a suicide rate higher than 24.0 per 100,000 individuals for fourteen consecutive years [1, 2]. “The Basic Act for Suicide Prevention”, the basic anti-suicide law, was established in 2006, and various suicide prevention measures were implemented and promoted in a complex, simultaneous manner [3–5]. For example, the policy included measures for raising public awareness, training gatekeepers, enhancing collaboration with private organizations, and establishing a medical care coordination system by which primary care providers can refer depressive patients to a psychiatrist [1, 2]. As a consequence, the number of annual suicides started to decrease across Japan, falling below 30,000 in 2012, with a relatively large decline in the suicide rate from 24.0 in 2011 to 21.8 in 2012 [2]. However, although the rate continued to decrease to 16.0 by 2019, it still remains the highest among the G7 countries [2]. It is also of concern that suicides in Japan will possibly increase again due to the COVID-19 pandemic [6, 7]. Since there have been few studies to provide an explanation for the suicide decline in Japan [8], it appears necessary to clarify the factors that contribute to the decrease in suicides in order to take appropriate suicide prevention measures in the future.

The mental health activity that promotes connecting individuals who need psychiatric treatment with adequate medical services is one of the major strategies of the national suicide prevention plans in Japan [2]. As one of the pillars of suicide prevention, the countermeasure of connecting untreated high-risk for suicide individuals with adequate psychiatric treatment was taken, thereby increasing the rate of psychiatric care for groups at high risk of suicide. Thus, improvement of the psychiatric treatment rate for high-risk suicide groups during the period of suicide decline may have contributed to their decrease in number in Japan. However, the effect of improved psychiatric treatment rates on suicide reduction has not been verified, as it is impossible to examine longitudinal changes in psychiatric treatment rates in suicide cases based on police reports or psychological autopsies in Japan in terms of viability [1, 9, 10].

Investigation of serious suicide attempters [11] is thought to be an alternative and promising methodology for exploring factors associated with suicidal behaviors [12]. At our hospital, Nippon Medical School (NMS) Hospital, we have lengthy and significant psychiatric intervention experience at the Critical Care Medical Center (CCM) for the most physically ill suicide attempters. Our CCM was established in 1977 as the first advanced emergency medical center in Japan. As one of the tertiary emergency medical facilities of the Tokyo Metropolitan Government, the center receives about 1600 to 1800 emergency patients per year, about 5% of whom are suicide attempters. We have reported the results of retrospective observational studies of the characteristics of serious suicide attempters among adolescents as well as of gender differences of precipitating factors for suicide attempts [13, 14].

From the annual report by the Fire and Disaster Management Agency (FDMA), the national statistics on ambulance transportation, the number of cases of ambulance transport due to self-harm peaked at 52,630 in 2009 and then steadily declined to 35,377 by 2017 [15]. It is estimated that the decrease in the number of serious suicide attempters is similar to that in the number of suicidal deaths, although reports on differences between the two have been lacking. A survey targeting suicide attempters would possibly confirm their psychiatric treatment status and assist in the investigation of the long-term changes of psychiatric treatment rates in the suicide high-risk group.

In the present study, we aimed to investigate whether the number of serious suicide attempters admitted to CCM of the NMS Hospital decreased in parallel with the number of suicides in Tokyo during the same period. We also aimed to examine whether the psychiatric treatment status of serious suicide attempters improved or not, as well as to explore any association between improvement of psychiatric treatment status in serious suicide attempters and a decrease in suicides during the same period and in the same region, that is, in Tokyo.

## Methods

### Data sampling

Study subjects were enrolled from the period between January 2006 and December 2017. We collected the number of suicides in the Tokyo area from an open source that is annually reported by the Metropolitan Police Department (MPD) [16]. In this study, we regarded 2012 as the turning point to a decline in suicides in Japan.

The subjects were patients who attempted suicide and required hospitalization at the CCM for at least 2 days, according to the criteria of “medically serious suicide attempters” [12] between January 2006 and December 2017. It was considered an actual “suicide attempt” if there was any intent/desire to die in association with the act, which is the same definition as that of the Columbia-Suicide Severity Rating Scale [17]. We collected data from the patients’ medical records retrospectively. We judged patients as being “under psychiatric treatment” if they had appointments or a history of psychiatric medical contact within 3 months before their suicide attempts.

We separated the subjects into groups of two terms, one of 6 years before the decline in suicide deaths (January 2006–December 2011), and the other of 6 years after the start of the decline (January 2012–December 2017). We defined the changes between the two terms as a factor of “period”.

Age was divided into four ranges, younger than or equal to 19 years (19 or younger), 20 to 39 years (20–39), 40 to 59 years (40–59), and equal to or older than 60 (60 or older).

Data were collected annually by age and gender for the number of suicide deaths, the number of suicide attempters, and the psychiatric treatment rates, respectively.

### Statistical analysis

For the numbers of suicide attempters and suicide deaths, differences by the factors of “period”, “age”, and “gender” were examined by analysis of variance (ANOVA). The main effect of the period was examined in terms of whether there was a difference between the number of attempters and the number of suicide deaths in the two periods before and after suicide reduction. For the items with a significant interaction, t-tests were performed to determine whether there was a difference in the periods of all of the age and gender subgroups.

Differences in the psychiatric treatment rate between genders according to the two periods were examined by chi-square test. In addition, differences in psychiatric treatment rates between the two periods according to gender, and between these subgroups, were examined by chi-square test, respectively.

Additionally, we combined subgroups that had changed their psychiatric treatment rates into a single group, and we analyzed the correlation between the annual treatment rate and the annual number of suicide deaths by Pearson’s correlation coefficient.

We used a significance level of *p* < 0.05 and two-sided probability. R version 4.0.2 statistical package was used for the entire analysis.

## Results

### Changes in suicides in Tokyo

**Fig. 1:**
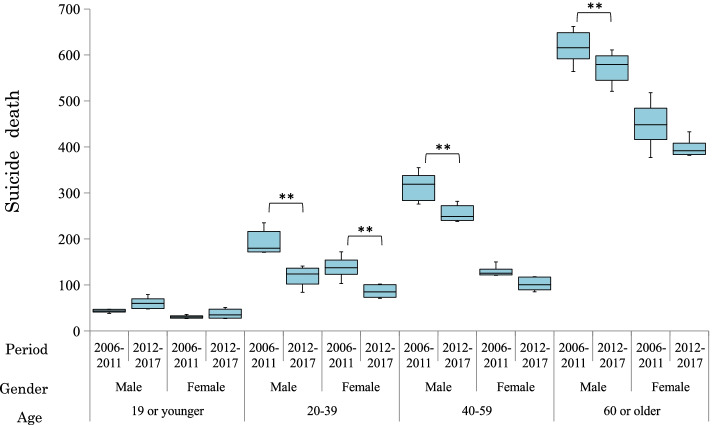
Changes in the number of suicides in Tokyo by period (age and gender subgroups). Footnote: 1. ** < .01; * < .05.; 2. Period: 2006–2011, 6 years before the decline in suicide deaths; (January 2006–December 2011); 2011–2017, 6 years after start of the decline (January 2012–December 2017); 3. Age: 19 or younger, younger than or equal to 19 years; 20–39, 20 to 39 years; 40–59, 40 to 59 years; 60 or older, equal to or older than 60

There were 32,783 suicides in Tokyo during the whole study period. 531 (2006–2011: 351; 2012–2017: 180) subjects with insufficient information regarding age were excluded. Finally, the numbers of suicide deaths included in the analysis were 17,364 (2006–2011) and 14,888 (2012–2017). According to ANOVA, a significant main effect of “period” was recognized, and the number of suicides in Tokyo decreased significantly (F(1,1) = 44.61, *p* < 0.01). There were significant interactions between period and age (F(1,3) = 7.31, *p* < 0.001) and between period and gender (F(1,1) = 9.13, *p* = 0.003). Significant decreases were observed in age and gender subgroups: males aged 20–39 (t(10) = 3.77, *p* = 0.004), males aged 40–59 (t(10) = 3.63, *p* = 0.005), males aged 60 or older (t(10) = 2.82, *p* = 0.018), and females aged 20–39 (t(10) = 4.21, *p* = 0.002) (Fig. 1).

### Changes in serious suicide attempters admitted to CCM

**Fig. 2:**
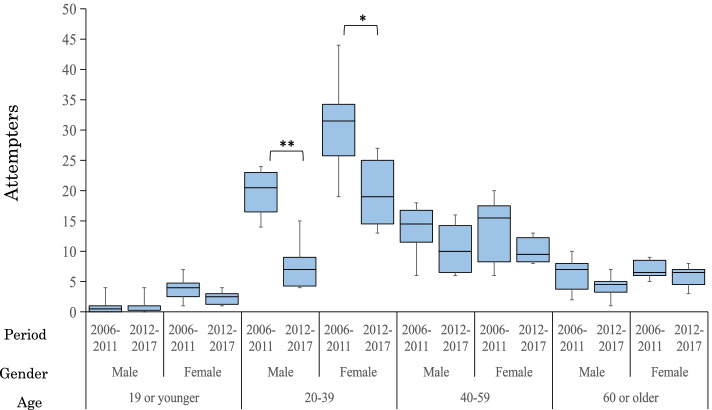
Changes in the number of suicide attempters in CCM by period (age and gender subgroups). Footnote: 1. ** < .01; * < .05; 2. Period: 2006–2011, 6 years before the decline in suicide deaths (January 2006–December 2011); 2011–2017, 6 years after start of the decline (January 2012–December 2017); 3. Age: 19 or younger, younger than or equal to 19 years; 20–39, 20 to 39 years; 40–59, 40 to 59 years; 60 or older, equal to or older than 60

During the whole study period, 21,271 patients were admitted to the CCM, and the number of suicide attempters included for the analysis was 942. The numbers of suicide attempters in 2006–2011 and 2012–2017 were 573 (5.0% of 11,452, total CCM inpatients) and 369 (3.8% of 9819), respectively. The results of ANOVA showed that there was a main effect of period, and the number of suicide attempters decreased significantly (F(1,1) = 26.20, *p* < 0.001). A significant interaction was found between period and age (F(1,3) = 9.05, p < 0.001) but not between period and gender (F(1,1) = 0.003, *p* = 0.96), and a significant decrease was observed only in the subgroup of those aged 20–39 (t(10) = 4.40, *p* = 0.001). Further, t-tests of changes by age and gender subgroups confirmed a significant decrease only in males aged 20–39 (t(10) = 4.89, *p* < 0.001) and females aged 20–39 (t(10) = 2.55, *p* = 0.029) (Fig. 2).

### Changes in psychiatric treatment rate in serious suicide attempters

There was a significant gender difference in the rate of psychiatric treatment. Before the suicide reduction, the rate was higher in females (69.5% in females and 44.6% in males) (χ^2^(1) = 35.70, *p* < 0.001), and after the suicide reduction, the rate was also higher in females (74.1% in females and 63.1% in males) (χ^2^(1) = 5.01, *p* = 0.025) (Table 1).

**Table 1:**
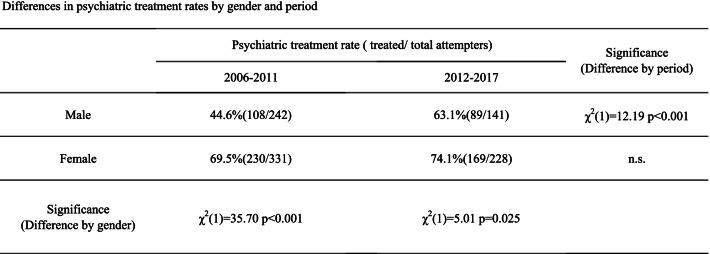
Differences in psychiatric treatment rates by gender and period

Footnote: The psychiatric treatment rates were defined as the percentages of attempters receiving psychiatric treatment among all attempters; 2006–2011, 6 years before the decline in suicide deaths (January 2006 to December 2011); 2011–2017, 6 years after start of the decline (January 2012 to December 2017).

When changes in psychiatric treatment rates were examined for the periods before and after the start of decrease in suicides, significant improvements in psychiatric treatment rates were found only in the male groups aged 20–39 (from 55.1 to 78.3%, χ^2^(1) = 7.52, *p* = 0.006) and 40–59 (from 39.5 to 65.1%, χ^2^(1) = 9.27, *p* = 0.003) (Table 2). When the rate of psychiatric treatment was examined in the group of adult males aged 20 to 59, the rate improved from 48.7% (97/199) to 70.6% (77/109) (χ^2^(1) = 13.74, *p* < 0.001).

**Table 2:**
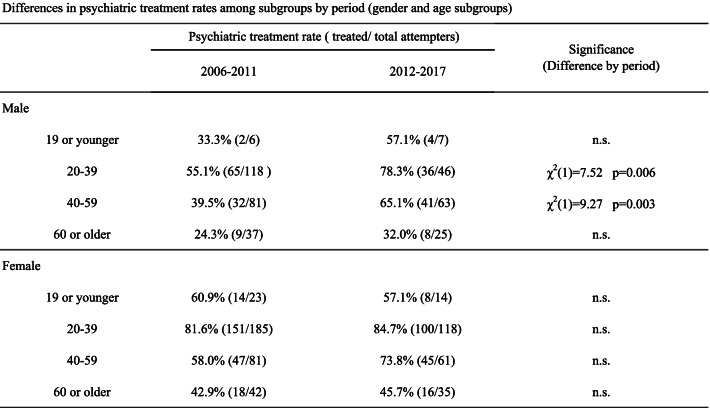
Differences in psychiatric treatment rates among subgroups by period (gender and age subgroups)

Footnote: The psychiatric treatment rates were defined as the percentages of attempters receiving psychiatric treatment among all attempters; 2006–2011, 6 years before the decline in suicide deaths (January 2006 to December 2011); 2011–2017, 6 years after the start of the decline (January 2012 to December 2017); 19 or younger, younger than or equal to 19 years; 20–39, 20 to 39 years; 40–59, 40 to 59 years; 60 or older, equal to or older than 60.

In the groups aged 60 years or older, the rate of psychiatric treatment was low before the decrease in suicides, at 24.3% in males and 42.9% in females, and remained low after the start of decrease in suicides, at 32.0% in males and 45.7% in females, with no improvement observed (Table 2).

### Correlation between psychiatric treatment rates in serious suicide attempters and numbers of suicides

**Fig. 3:**
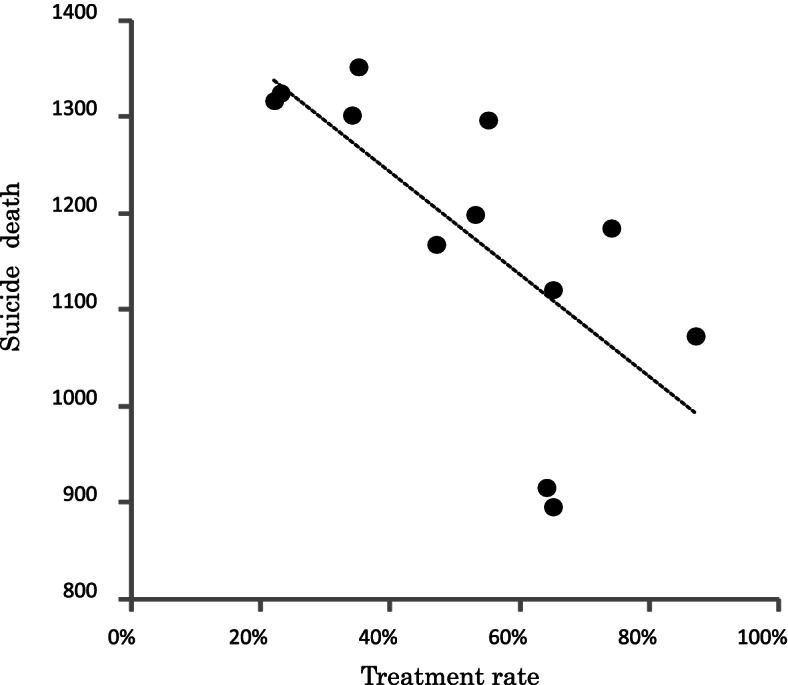
Yearly scatter plot of psychiatric treatment rate and the number of suicide deaths (males 20–59). Footnote: The psychiatric treatment rates were defined as the percentages of attempters receiving psychiatric treatment among all male attempters aged 20–59

The correlation between psychiatric treatment rates and numbers of suicides was examined in the group of males aged 20–59 years who showed improvement of the psychiatric treatment rate in suicide attempters. There was a significant negative correlation between psychiatric treatment rates and the number of suicides (*r* = − 0.59, *p* = 0.042) (Fig. 3). In other words, improvement in the rate of psychiatric treatment was significantly correlated with the decrease in the number of suicides.

## Discussion

### Decrease of serious suicide attempters admitted to CCM

Similar to the decrease in the number of suicides in the Tokyo area, the number of serious suicide attempters who were admitted to the CCM also decreased. There was a significant decrease in the number of suicide attempters especially in the age group of 20–39 years for both genders, just as there was a significant decrease in suicides. However, whereas the number of suicides decreased significantly in males aged 40 and older, the number of suicide attempters did not change significantly in either gender. Regarding such discrepancies, it is known that suicide is more common among middle-aged and older males and attempted suicide is more common among young females in Japan [1, 2]. In this study, as in previous reports, the number of suicides was higher among older adults and was more male-dominant, whereas the number of suicide attempters was greater among young adults aged 20–39 and was more female-dominant. Compared to suicide deaths, the number of suicide attempters was relatively low among men and the elderly. This may explain why there was no decrease in the number of attempted suicides among men aged 40 and older.

### Improvement of psychiatric treatment rate among serious suicide attempters

The purpose of the present study was to examine whether psychiatric treatment rates improved in a high-risk suicide population during the period of suicide decline. Comparing psychiatric treatment rates of serious suicide attempters admitted to CCM before and after a decrease in suicides, we confirmed that there was a significant improvement in psychiatric treatment rates from 48.7 to 70.6% in the group of males aged 20–59 years. In contrast, no significant improvement was observed in the female group, but this may have been due to their originally high rate of psychiatric treatment. Psychological autopsy studies in Japan reported that 44.1 to 50% of individuals who died by suicide were treated by psychiatrists [9, 10]. Psychiatric treatment rates for those who committed suicide have been lower than those for attempted suicides during the period of decline. Longitudinal studies of psychiatric treatment rates among suicides are not available, but psychiatric treatment rates of those among suicide deaths may have increased during periods of declining suicides.

### Improvement in psychiatric treatment rate and a decrease in the number of suicides

Interestingly, in the group of males aged 20–59 years, improvement in psychiatric treatment rates correlated with a decrease in the number of suicides during the same period. This result suggests that improved psychiatric treatment rates among males in this age group may have contributed to the reduction in the number of suicides in Tokyo. There is considerable evidence that measures for connecting untreated populations in need of treatment with psychiatric care are effective in preventing suicide [18, 19]. Several psychiatric treatments, such as pharmacotherapy and cognitive behavioral therapy, have been shown to be effective in preventing suicidal behavior [20–24]. A high rate of psychiatric consultation may indicate the severity and persistence of psychopathology among serious suicide attempters and/or that mental health services are being adequately provided. Additionally, contact with mental health professionals may have mitigated the lethality of the attempts. On the other hand, the number of suicides is still high, and further efforts should be made. There is a need to further improve treatment to prevent suicide for those who come under psychiatric care. For example, when a psychiatric healthcare provider identifies a patient at risk for suicide, it may be necessary to coordinate the patient’s access to problem-solving support (e.g. financial, legal and employment support), and to provide care in collaboration with support groups. It will be important for psychiatric institutions that identify such patients to provide case management [24] and to work with support groups to prevent suicide.

Together with the results of this study, there is a possibility that suicidal behavior decreased in conjunction with improvement in the psychiatric treatment rate. On the other hand, the elderly group with the highest number of suicides in both males and females had the lowest rate of receiving psychiatric treatment, and they showed no improvement in their psychiatric treatment rate. The elderly population consists of those who are more prone to physical illness and suicide risks such as loneliness and lack of social support, and lifestyle changes due to the COVID-19 pandemic may also be involved in increasing some of these problems [25]. These results indicate that those at high risk for suicide among the elderly population may be receiving insufficient psychiatric care, and in fact this issue may require urgent attention.

Limitations of the current study include the fact that we used data on suicide cases from the whole area of Tokyo. Although suicide attempters are transported to our emergency department from a broad area, this does not cover the whole Tokyo area. So, these study fields do not exactly match. Our study was conducted as a single-site survey. This will also be a limitation of the data representativeness when it is used as a reference for taking future suicide countermeasures. Thus, surveys in a broader area and multicenter collaborative studies with larger numbers of subjects will be necessary to solve these limitations. In this survey, although we focused on the relationship between the psychiatric treatment rate and the number of deaths by suicide, the contributions of other psychosocial factors and economic factors that have been indicated as also being related to suicide were not examined. Furthermore, we did not include variables related to the subject, such as the history of suicide attempts or their means. The addition of these data to the analysis may allow a more detailed description of the phenomenon of suicidal behaviour.

## Conclusions

In conclusion, the number of serious suicide attempters admitted to the CCM decreased together with the number of suicides in Tokyo. Especially in the age group of 20–39 years, the number of serious suicide attempters of both genders decreased, as did the number of suicides. The results of this study confirmed that there was significant improvement in psychiatric treatment rates in the severe suicide attempt group of males aged 20–59 years. In addition, improvement in the psychiatric treatment rate correlated with a decrease in the number of suicides of the same gender and age in the community.

These results suggest that the improvement of psychiatric treatment rates in high-risk suicide groups may have contributed to the reduction of suicides in Tokyo. However, the rate of psychiatric treatment in the elderly group, which has the highest number of suicides among both males and females, has remained low and has not improved during the period of suicide decline, suggesting a future issue needing to be addressed for suicide prevention.

## Data Availability

The datasets generated and/or analyzed during the current study are not publicly available due to the used data protection declaration, but are available from the corresponding author on reasonable request.

## Abbreviations

ICD: International Statistical Classification of Diseases and Related Health Problems
